# Overcoming Obstacles

**DOI:** 10.5935/1678-9741.20150083

**Published:** 2015

**Authors:** Domingo M. Braile

**Affiliations:** 1 Editor-in-chief - BJCVS.

In times of economic crisis, with the government cutting funds in various sectors, including science and technology, with serious consequences in scientific research and, consequently, the production of good articles, periodicals must learn to reinvent themselves in order to be most read/accessed by attracting more interesting studies. To make matters worse, the difficult moment that crosses the country makes that Brazil is no longer an attractive market even for science, without obtaining funds from international institutions for the development of research with sufficient potential to develop into good standard original articles.

Linking this situation to the *modus operandi* of the Brazilian and international science, in which the impact factor of a journal ends up being one of the main attractions, but the principal, for an author to submit his study to a particular journal, those who know how to unite quality and creativity are more likely to get along in this "race for good articles".

It is not an easy task for Brazilian publications in general.

While the international journal with the highest impact factor (IF), according to data from the Journal Citation Reports, from Thomson-Reuters, 2015, the CA: A Cancer Journal for Clinicians, has an IF of 115.840, followed by New England Journal of Medicine, with "only" 55.873, the Brazilian journal with a higher IF, the Diabetology & Metabolic Syndrome, achieved 2,173 index. It means, an abysmal distance^[1]^.

We feel a reflection of this cooling situation on the interest in Brazilian scientific production also in the Brazilian Journal of Cardiovascular Surgery (BJCVS), whose site that has come to be accessed by more than 100 countries in the recent past, had this number reduced to almost one-third, even with an intense work on the disclosure of the journal and many actions to attract more readers and authors from abroad, using only the English language throughout its structure, including the articles, by recognizing this as the "lingua franca" of scientific communication.

The reduction, fortunately, was not reflected in the number of articles submitted from abroad. Just as an example, in this issue we have studies produced in the China, Iran, Turkey, and USA. But this scenario is a warning to intensify the search for more readers and more manuscripts, so we can grow again in FI and Qualis, from Capes.

We are taking important steps to ensure that the BJCVS can approach competitively to potential readers and authors, drawing attention to our values, as being our journal the only publication of the specialty in the Southern Hemisphere, completing 30 years of fruitful existence.

We hope in this way to present again a substantial growth in the audience of our website and offering of impactful articles, which will be reflected in the creation of a virtuous circle of increased other articles and more citations.

After all, since the beginning of its history the BJCVS was able to overcome obstacles. As informed earlier, the 43^rd^ Brazilian Congress of Cardiovascular Surgery, which will take place from 7 to 9 April 2016, in Fortaleza, will be part of its agenda focusing on the anniversary of BJCVS.

The Instructions for Authors (http://www.bjcvs.org/page/6) are continuously being updated in order to meet more adequately the needs of authors and reviewers, still giving more transparency to the process of submission and review of a study, always emphasizing the importance of the rigorous "peer-review" process.

We now make available the forms used in the reviews in BJCVS in order to the authors, when submitting their studies, can know in advance the criteria to be used in the evaluation of his manuscript, item by item, thus allowing prepare their study more accurately and are accepted in a shorter period for "ahead of print' publication and depending on its intrinsic value, being part of the nearest periodic edition.

We also provide a new link to the MeSH on Demand (https://www.nlm.nih.gov/mesh/MeSHonDemand.html), a very useful tool that allows, when inserting the abstract in a specific field, get after a few seconds, a list of suggestions of descriptors that can be used to enrich in number and quality the key words, a key factor for the article to be found by those who do searches in any of the databases.

It is a great help to the authors, even those more experienced in choosing the descriptors to be used in the study.

## Social networks

Social networks are becoming increasingly important in day-to-day life of people and institutions, including the scientific area which also has adhered to this form of communication. Our Facebook (https://www.facebook.com/bjcvs) was streamlined and now has been updated frequently.

It was also opened an account on Twitter (twitter.com/bjcvs) for short notes.

In addition, we created a blog (http://bjcvs.blogspot.com.br/), in which I ask you to navigate and help us.

They are precious spaces to disseminate news, information and discussions on cardiovascular surgery and related fields and also on scientific communication. For not being a scientific jour-nal the language may be more colloquial, fleeing the necessary scientific rigor of the language of the BJCVS. The texts will be posted in Portuguese, without concern for size. We invite you to publish your ideas and comments.

We have established a new platform for the flip version of the journal, lighter and interactive, facilitating navigation and reading.

The BJCVS applications for smartphones mobile devices in the Android and iOS operating systems are still available for download for free. Use them and broadcast their existence.

## New e-mail

The BJCVS has new contact e-mail: bjcvs@sbccv.org.br

Questions, suggestions and criticisms can be sent via this new email address and will be evaluated and met with attention ever given by our Editorial Board.

## CME

We make available the following articles for the testing of Continuing Medical Education (CME) in this issue: "*Experimental Study and Early Clinical Application of a Sutureless Aortic Bioprosthesis" (page 515); "Evaluation of Pulmonary Reperfusion Injury in Rats Undergoing to Mesenteric Ischemia and Reperfusion and Protective Effect of Postconditioning on this Process" (page 533); "Adjunctive Hyperbaric Oxygen Therapy or Alone Antibiotherapy? Methicillin Resistant Staphylococcus aureus Mediastinitis in a Rat Model" (page 538); and "Factors Associated With the Development of Chronic Post-Sternotomy Pain: a Case-Control Study" (page 552)*. I remember that the CME is a great way to update the knowledge and became worth 5 points in the title Brazilian Society of Cardiovascular Surgery Proof of Title. We are open to suggestions and criticisms to improve the system.

My warmest regards! Domingo M Braile ^1^Editor-in-chief - BJCVS
